# Bursting dynamics in the normal and failing hearts

**DOI:** 10.1038/s41598-017-05198-z

**Published:** 2017-07-19

**Authors:** Vladimir E. Bondarenko, Andrey L. Shilnikov

**Affiliations:** 1 Department of Mathematics and Statistics and Neuroscience Institute, Georgia State University, 30 Pryor Street, Atlanta, GA 30303 United States; 2 Institute for Information Technologies, Mathematics and Mechanics, Nizhni Novgorod State University, Gagarin Av. 23, 606950 Nizhni Novgorod, Russia

## Abstract

A failing heart differs from healthy hearts by an array of symptomatic characteristics, including impaired Ca^2+^ transients, upregulation of Na^+^/Ca^2+^ exchanger function, reduction of Ca^2+^ uptake to sarcoplasmic reticulum, reduced K^+^ currents, and increased propensity to arrhythmias. While significant efforts have been made in both experimental studies and model development to display the causes of heart failure, the full process of deterioration from a healthy to a failing heart yet remains deficiently understood. In this paper, we analyze a highly detailed mathematical model of mouse ventricular myocytes to disclose the key mechanisms underlying the continual transition towards a state of heart failure. We argue that such a transition can be described in mathematical terms as a sequence of bifurcations that the healthy cells undergo while transforming into failing cells. They include normal action potentials and [Ca^2+^]_i_ transients, action potential and [Ca^2+^]_i_ alternans, and bursting behaviors. These behaviors where supported by experimental studies of heart failure. The analysis of this model allowed us to identify that the slow component of the fast Na^+^ current is a key determining factor for the onset of bursting activity in mouse ventricular myocytes.

## Introduction

Heart failure has been the focus of intensive experimental studies, along with mathematical modeling efforts, for a long time. This has been for the purpose of disclosing heart failure’s causes and to propose its effective remedies. In failing hearts, several major cellular abnormalities, identified empirically, are considered indicative of forthcoming heart failure. Among them are: reduced intracellular [Ca^2+^]_i_ transients due to increased function of the Na^+^/Ca^2+^ exchanger and reduced function of the SERCA pump, and downregulation of the K^+^ repolarizing currents, all resulting in a larger propensity of arrhythmias^1, 2^. Significant investigation efforts were undertaken to explore and justify transgenic mice as the experimental animal models for heart failure. Some of the mouse models die at early development stages due to severe changes in structures of the mouse heart and/or insufficient metabolism, whereas other mice survive and live until adulthood, demonstrating the advantages or disadvantages of the upregulation or downregulation of particular protein functions^3^. Many surviving transgenic mice become more susceptible to such cardiac arrhythmias as tachycardia and fibrillation. In the majority of transgenic mice, the arrhythmia has a re-entrant nature^4^, while some non-re-entrant arrhythmias were reported too^5^.

In this computational paper, we employ a comprehensive/highly detailed mathematical model of mouse cardiac cells that was previously developed for wild type (WT) and transgenic (TG) mice overexpressing TNF-α (mouse model of the heart failure)^6, 7^. The model supported the validity of the key cellular abnormalities of the failing heart described above^7^. Despite a diversity of mathematical models of the failing ventricular myocytes^7–9^, approaches aimed at modeling transition stages from normal to failing cardiac cells remain yet undeveloped.

The objective of this research is to narrow this gap with a novel concept developed for the detailed understanding of morph transitions from healthy to failing cardiac cell functions. These transitions are interpreted in the form of bifurcation sequences that the degrading oscillatory dynamics of cells go through as key parameters of the most vital characteristics and the state of the heart are continually changed. As guiding examples, we consider two mathematical models proposed for ventricular cells from WT and TG mice^7^ that differ in seven key parameters, including Ca^2+^ release rate from the sarcoplasmic reticulum (SR), v_3_, Na^+^/Ca^2+^ exchanger rate, k_NaCa_, conductance of the fast recovering component of the transient outward K^+^ current, I_Kto,f_, conductance of the ultrarapidly activating K^+^ current, I_Kur_, and three parameters of the time-independent inward rectifier K^+^ current, I_K1_, (see Supplementary Table 1). We showed that transient behaviors of the action potential and [Ca^2+^]_i_ transient, along with interspike intervals between consequent potentials generated by mouse ventricular myocytes change qualitatively and quantitatively through the stages separating normal behaviors, alternans, and bursting activity. The proposed approach can be used for investigations of other mouse ventricular cell models describing transitions between normal and pathological states, as this specie is widely used in experimental models of heart failure. The method can also be used in the field of cardiac optogenetics, when the expressed channels in the mouse heart generates almost constant inward currents upon optical illumination^10, 11^.

## Methods

**Figure 1:**
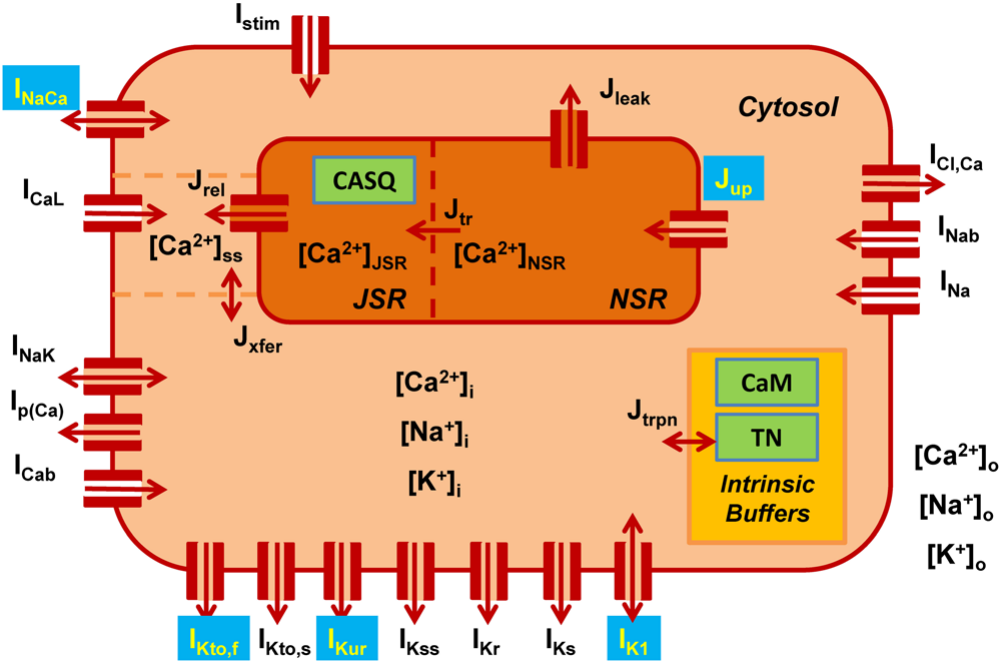
Schematic diagram of the mouse ventricular myocyte model that includes ionic currents and Ca^2+^ fluxes. Transmembrane currents are: I_Na_, the fast Na^+^ current, I_CaL_, the L-type Ca^2+^ current, I_p(Ca)_, the sarcolemmal Ca^2+^ pump, I_NaCa_, the Na^+^-Ca^2+^ exchanger, I_Kto,f_, the rapidly recovering transient outward K^+^ current, I_Kto,s_, the slowly recovering transient outward K^+^ current, I_Kr_, the rapid delayed-rectifier K^+^ current, I_Kur_, the ultra-rapidly activating delayed-rectifier K^+^ current, I_Kss_, the non-inactivating steady-state voltage activated K^+^ current, I_K1_, the time-independent K^+^ current, I_Ks_, the slow delayed-rectifier K^+^ current, I_NaK_, the Na^+^-K^+^ pump, I_Cl,Ca_, the Ca^2+^-activated chloride current, I_Cab_ and I_Nab_, the Ca^2+^ and Na^+^ background currents. I_stim_ is the external stimulation current. The Ca^2+^ fluxes within the cell are: J_up_ (uptake Ca^2+^ from the cytosol to the network SR), J_rel_ (Ca^2+^ release from the junctional SR), J_tr_ (Ca^2+^ flux from the network to junctional SR), J_leak_ (Ca^2+^ leak from the SR to the cytosol), J_xfer_ (Ca^2+^ flux from the subspace volume to the bulk myoplasm), J_trpn_ (Ca^2+^ flux to troponin). The model also includes Ca^2+^ buffering by troponin and calmodulin in the cytosol and by calsequestrin in the SR. The differences between WT and TG mice are highlighted in light blue color. They include I_Kto,f_, I_Kur_, I_NaCa_, J_up_, and I_K1_ (See details in Supplementary Table 1).

We employed mathematical models of isolated mouse ventricular myocyte for WT and TG mice^6, 7^, which schematic diagram is shown in Fig. 1. The model includes the fast Na^+^ current, I_Na_, the L-type Ca^2+^ current, I_CaL_, the sarcolemmal Ca^2+^ pump, I_p(Ca)_, the Na^+^-Ca^2+^ exchanger, I_NaCa_, the rapidly recovering transient outward K^+^ current, I_Kto,f_, the slowly recovering transient outward K^+^ current, I_Kto,s_, the rapid delayed-rectifier K^+^ current, I_Kr_, the ultra-rapidly activating delayed-rectifier K^+^ current, I_Kur_, the non-inactivating steady-state voltage activated K^+^ current, I_Kss_, the time-independent K^+^ current, I_K1_, the slow delayed-rectifier K^+^ current, I_Ks_, the Na^+^-K^+^ pump, I_NaK_, the Ca^2+^-activated chloride current, I_Cl,Ca_, the Ca^2+^ and Na^+^ background currents I_Cab_ and I_Nab_, and the external stimulus current, I_stim_. The model also includes comprehensive description of Ca^2+^ dynamics that contain the following Ca^2+^ fluxes: J_up_ (uptake Ca^2+^ from the cytosol to the network SR), J_rel_ (Ca^2+^ release from the junctional SR), J_tr_ (Ca^2+^ flux from the network to junctional SR), J_leak_ (Ca^2+^ leak from the SR to the cytosol), J_xfer_ (Ca^2+^ flux from the subspace volume to the bulk cytosol), J_trpn_ (Ca^2+^ flux to troponin). The Ca^2+^ buffering is done by troponin and calmodulin in the cytosol and by calsequestrin in the SR.

We investigated two different stimulation protocols of the model cells. First is the standard protocol, which stimulates cardiac cells with a sequence of small pulses of current, I_stim_ = 60 pA/pF with pulse duration τ_stim_ = 1 ms, at different basic cycle lengths (BCL) (periodic stimulation). In addition, we employed a new stimulation protocol using constant stimulus currents of the amplitude I_stim_, ranging from 0.0 to 1.0 pA/pF (steady-state stimulation). In the latter case, the model becomes an autonomous dynamical system with rich and complex dynamics compared to mouse ventricular myocytes at the standard protocol. To compare and characterize different cellular activities, we analyzed the behavior of the action potential, intracellular [Ca^2+^]_i_ transients, and interspike intervals.

## Results

**Figure 2:**
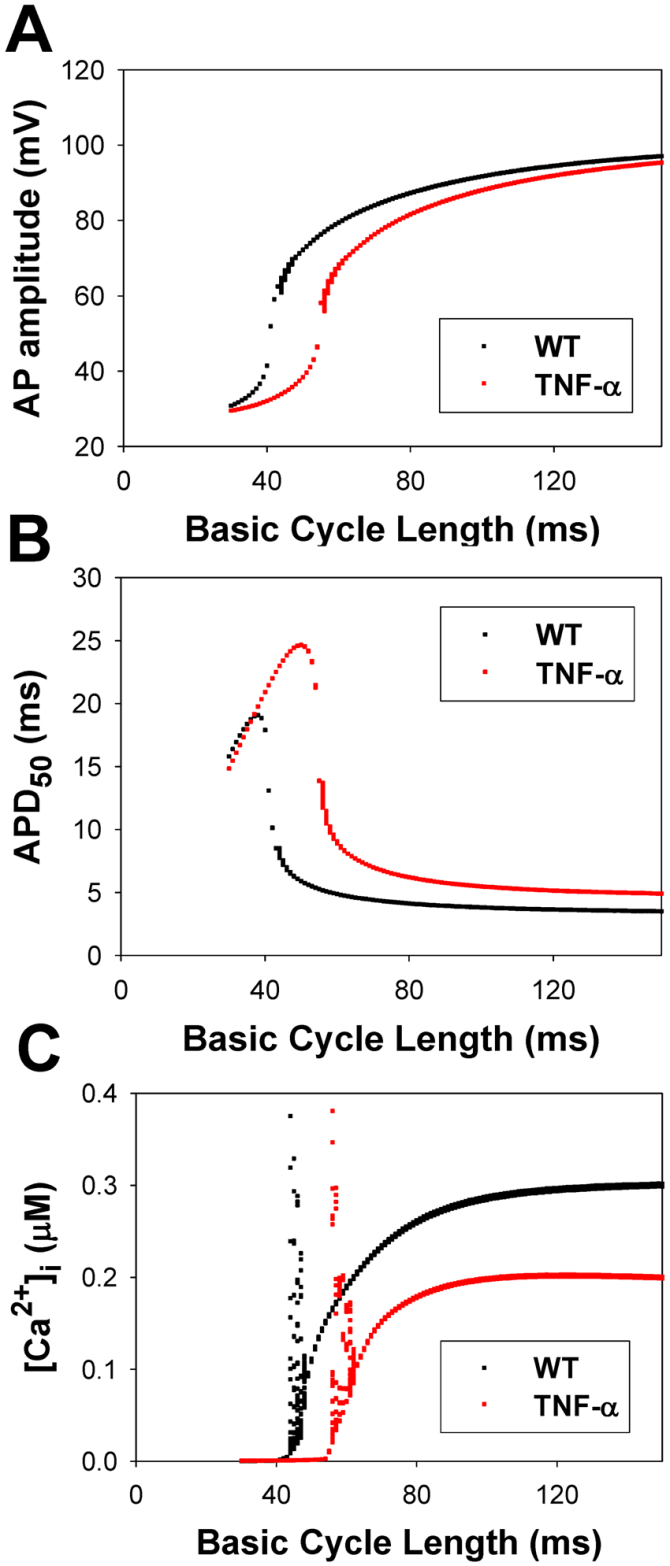
Simulated frequency dependence of AP amplitude (Panel A), AP duration at 50% of repolarization (Panel B), and [Ca^2+^]_i_ transients (Panel C) for WT (black squares) and TG (red squares) mouse ventricular myocytes at periodic stimulation. Basic cycle length was varied from 30 to 150 ms. AP amplitude, AP durations, and [Ca^2+^]_i_ transients were calculated in time interval from 48 to 50 s. I_Na_ with slow inactivation.

**Figure 3:**
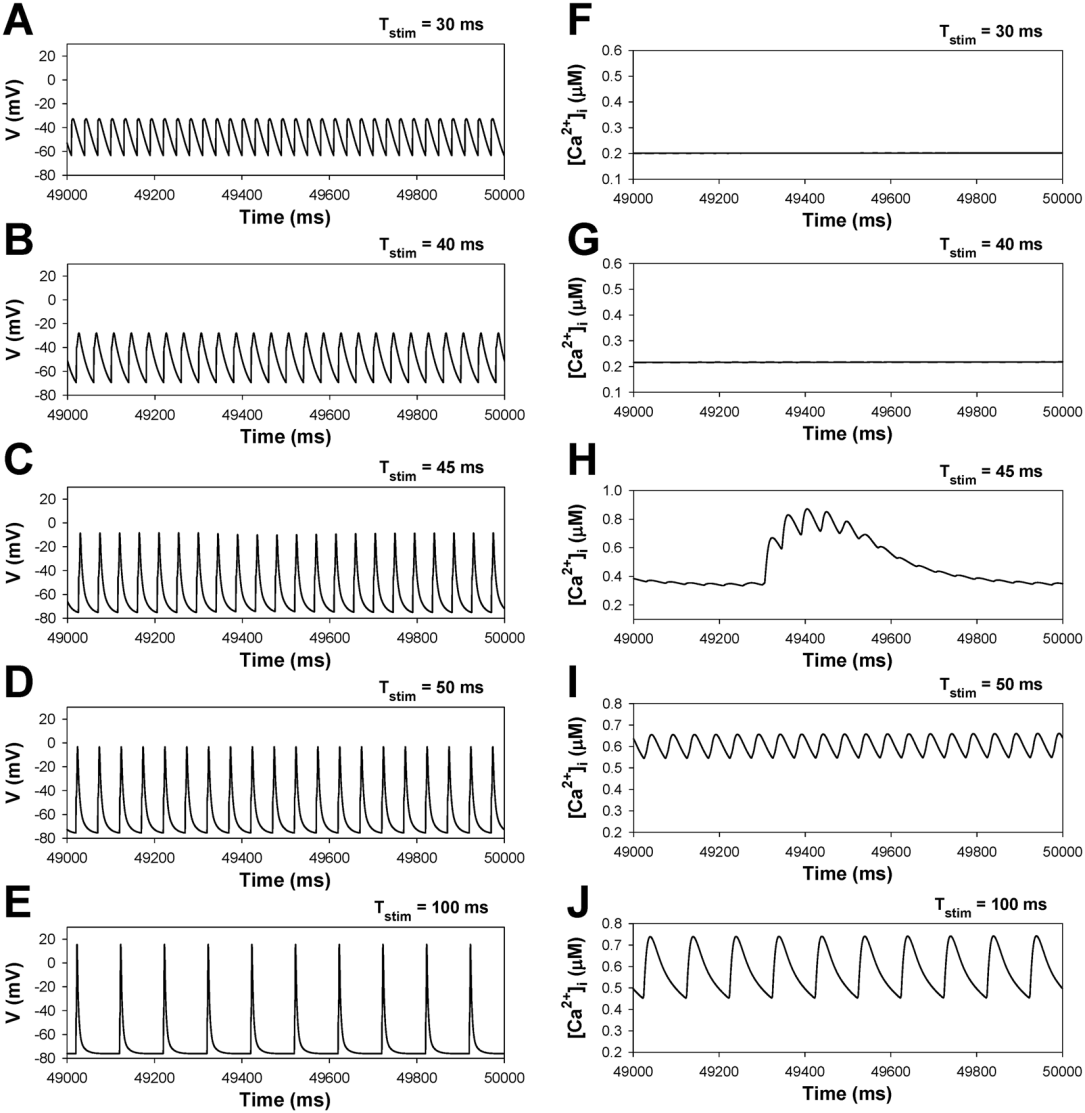
Simulated time series of AP (Panels A–E) and [Ca^2+^]_i_ transients (Panels F–J) in characteristic points of bifurcation diagrams for WT mouse ventricular myocytes in Fig. 2 during periodic stimulation: T_stim_ = 30 ms (Panels A,F); T_stim_ = 40 ms (Panels B,G); T_stim_ = 45 ms (Panels C,H); T_stim_ = 50 ms (Panels D,I); T_stim_ = 100 ms (Panels E,J). Bursting [Ca^2+^]_i_ transients are observed at T_stim_ = 45 ms (Panel H). I_Na_ with slow inactivation.

**Figure 4:**
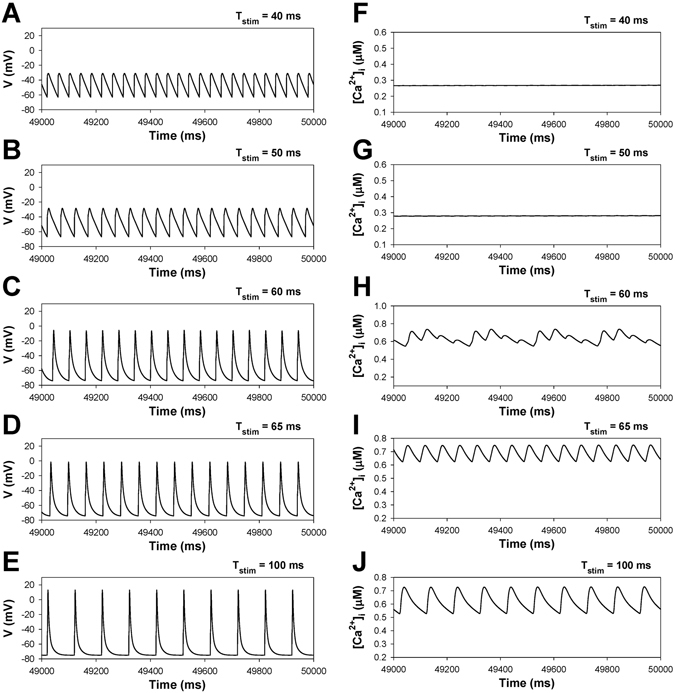
Simulated time series of AP (Panels A–E) and [Ca^2+^]_i_ transients (Panels F–J) in characteristic points of bifurcation diagrams for TG mouse ventricular myocytes in Fig. 2 during periodic stimulation: T_stim_ = 40 ms (Panels A,F); T_stim_ = 50 ms (Panels B,G); T_stim_ = 60 ms (Panels C,H); T_stim_ = 65 ms (Panels D,I); T_stim_ = 100 ms (Panels E,J). Bursting [Ca^2+^]_i_ transients are observed at T_stim_ = 60 ms (Panel H). I_Na_ with slow inactivation.

Figure 2 shows the dependence of the action potential amplitudes, action potential duration at 50% of repolarization, and peak [Ca^2+^]_i_ transients during standard pulsed stimulation of the basic cycle length for WT and TG mice. Figures 3 and 4 demonstrate the time-evolution of the action potentials and [Ca^2+^]_i_ transients at the specific characteristic values of BCL selected from Fig. 2. In WT mice (Fig. 2, black squares), one can observe highly irregular dynamics of [Ca^2+^]_i_ transients within a narrow region of BCL values, between 44 and 48 ms. As the BCL is increased from 30 to 100 ms, the action potential demonstrates nearly periodic activity (Fig. 3A–E), while [Ca^2+^]_i_ transients evolve from a steady-state level at BCL = 30 ms (Fig. 3F), small perturbations at BCL = 40 ms (Fig. 3G), bursting at BCL = 45 ms (Fig. 3H), followed by periodic activity (Fig. 3I,J). Note that [Ca^2+^]_i_ transients would be expected to affect AP dynamics through inactivation of the L-type Ca^2+^ currents. However, despite large magnitude bursting in [Ca^2+^]_i_ transients, this only gives rise to very small perturbations in APs, as seen from a comparison of Panels A and B in Fig. 2. This effect is explained by the fact that the L-type Ca^2+^ current is relatively small in mice as compared to other ionic currents, ~10 pA/pF, while two other repolarization currents, I_to_ and I_Kur_, are much larger, ~20–25 pA/pF each. Therefore, in mice, I_CaL_ and [Ca^2+^]_i_ cause a much smaller effect on the shape of the action potential. In addition, I_CaL_ inactivates much less due to Ca^2+^-dependent inactivation during short action potential (APD_50_ ~ 4.5 ms). Therefore, in mice, we do not observe the dramatic effects of I_CaL_ that are evident in the action potentials of larger species. Similar behaviors with wider BCL intervals for irregular activity were also observed for the model representing TG mice overexpressing TNF-α (Fig. 2, red squares). Highly irregular dynamics of [Ca^2+^]_i_ transients in TG mice are observed within BCL range from 56 to 62 ms. When BCL increases from 30 to 100 ms, the action potential of TG mice shows almost periodic activity (Figs 2 and 4A–E), while [Ca^2+^]_i_ transients changes from a steady-state level at BCL = 40 ms (Fig. 4F), small perturbations at BCL = 50 ms (Fig. 4G), bursting at BCL = 60 ms (Fig. 4H), followed by periodic activity (Fig. 4I and J). While WT and TG mice demonstrate similar activities with an increase of stimulation period, TG mice show more prolonged action potentials, higher diastolic [Ca^2+^]_i_, and smaller [Ca^2+^]_i_ transients as compared to WT mice (Figs 3 and 4).

**Figure 5:**
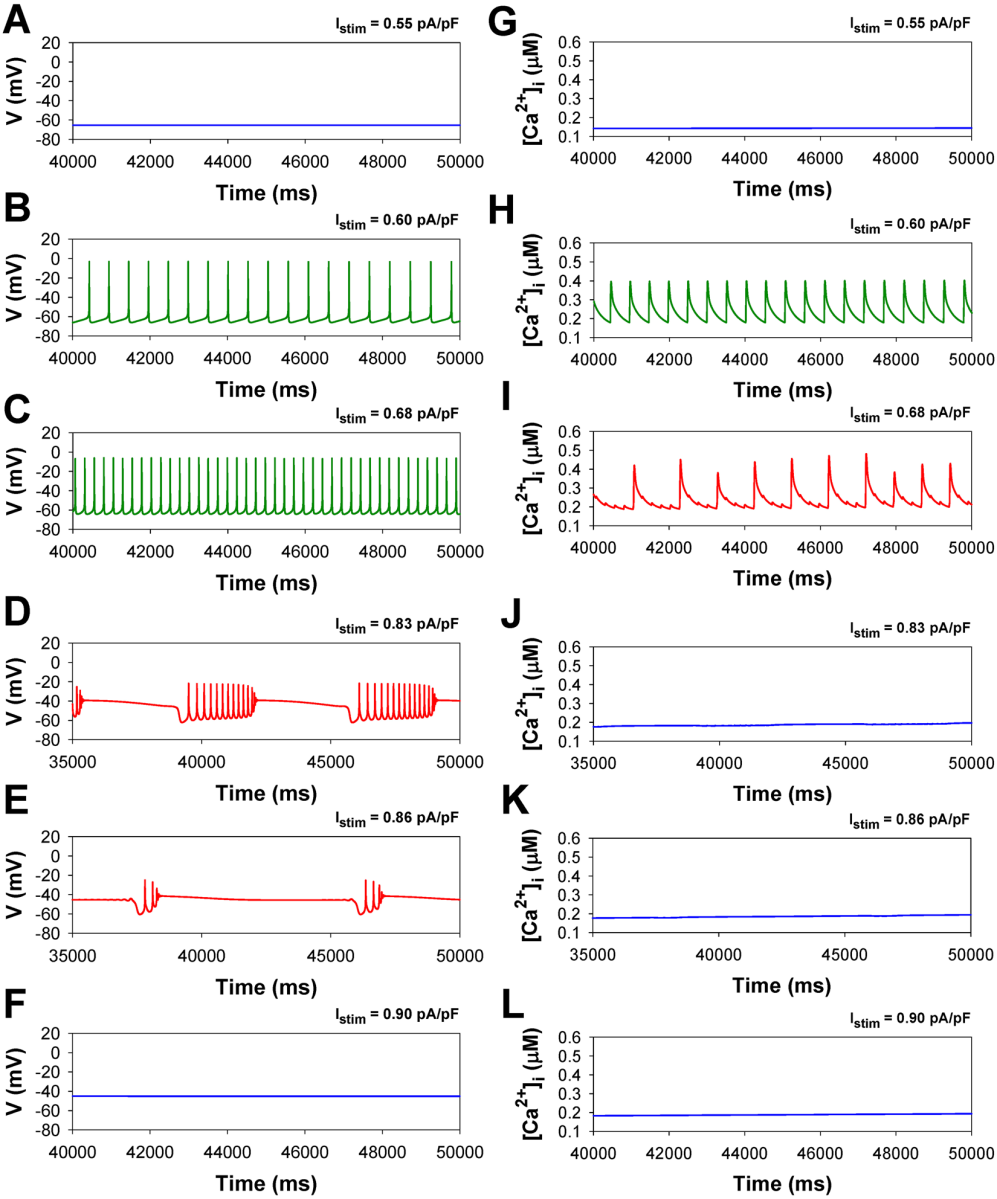
Simulated time series of AP (Panels A–F) and [Ca^2+^]_i_ transients (Panels G–L) in characteristic points of bifurcation diagrams for WT mouse ventricular myocytes in Fig. 6 during injection of continuous current: I_stim_ = 0.55 pA/pF (Panels A,G); I_stim_ = 0.60 pA/pF (Panels B,H); I_stim_ = 0.68 pA/pF (Panels C,I); I_stim_ = 0.83 pA/pF (Panels D,J); I_stim_ = 0.86 pA/pF (Panels E,K); I_stim_ = 0.90 pA/pF (Panels F,L). I_Na_ with slow inactivation.

Figure 5 demonstrates exemplary action potentials generated by the model of WT mouse ventricular myocytes using the second stimulation protocol, with steady-state stimulus currents (steady state stimulation). The stimulus current, in the form of a current step with different amplitudes (from 0 to 1.0 pA/pF), is applied at time moment t = 20 ms to the isolated model cell in the resting state. For I_stim_ less than 0.58 pA/pF, the cellular responses are transient voltage perturbations (not shown) that rapidly converge to a new steady state with a small depolarization (Fig. 5A). When I_stim_ exceeds a threshold value of 0.58 pA/pF the cell becomes excitable and generates an infinite series of action potentials (spiking) (shown in Fig. 5B and C). Further increase in I_stim_ through 0.79 pA/pF leads to the onset of a new type of activity where trains of rapid spiking with decreasing amplitudes, alternates with long intervals of silence/quiescence with depolarization decreasing from −40 to −50 mV (Fig. 5D and E). This study is the first report of the occurrence of such bursting activity in all models of ventricular myocytes. In the given WT mouse ventricular cell model, pronounced voltage bursting stably persists for a noticeable interval of I_stim_ ranging between 0.79 and 0.88 pA/pF. Note that the burst duration decreases with the increase of the stimulus current’s amplitude. When I_stim_ exceeds 0.88 pA/pF, the cell’s dynamics settle down on a steady state with relatively large depolarization (~ −45 mV) (Fig. 5F).

**Figure 6:**
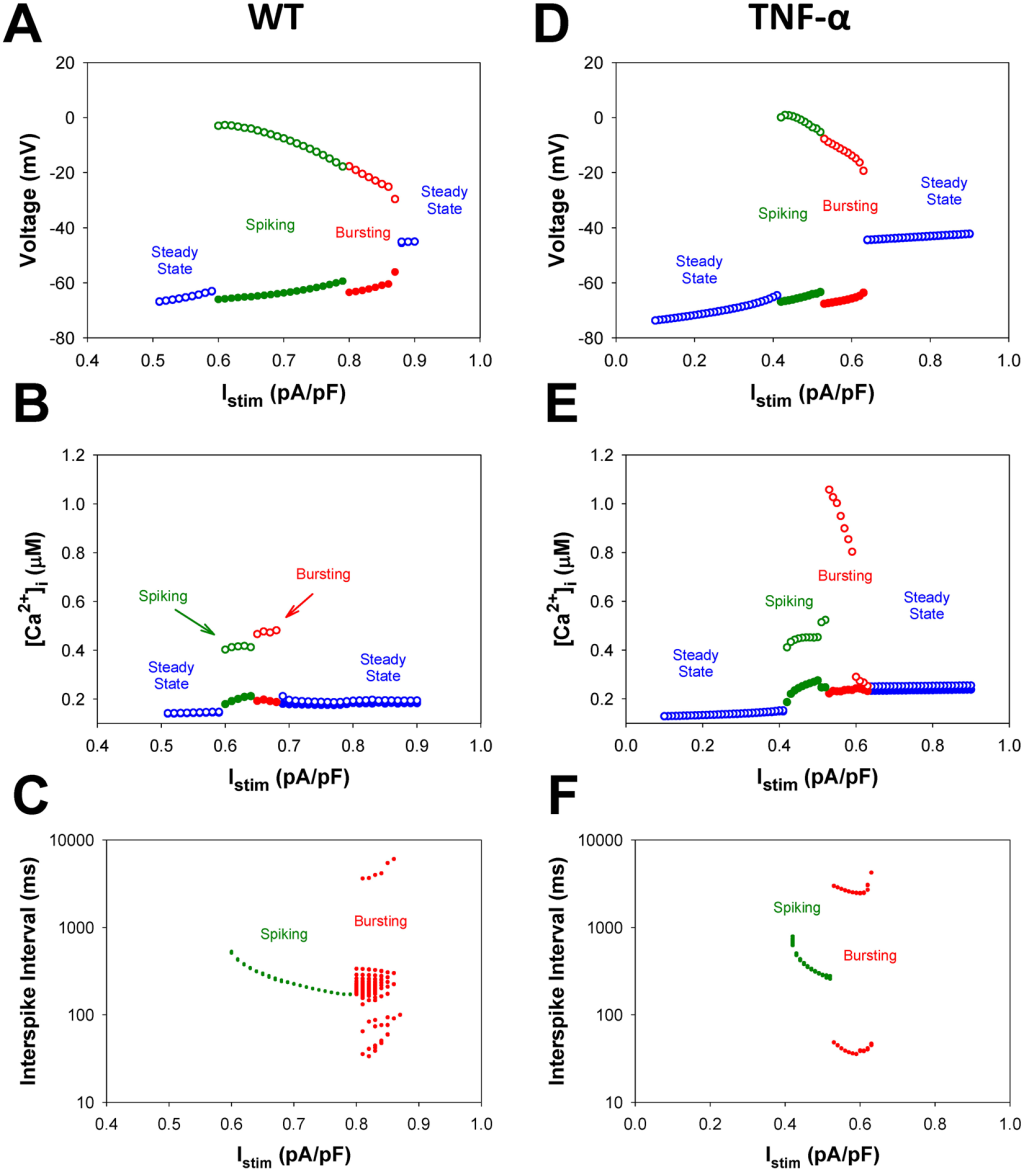
Simulated dependences of AP amplitudes (Panels A,D), [Ca^2+^]_i_ transients (Panels B,E), and interspike intervals (Panels C,F) for mouse ventricular myocytes on steady-state injected current I_stim_. Data for WT and TNF-α overexpressing mice are shown in Panels A–C and D–F, respectively. AP amplitude, [Ca^2+^]_i_ transients, and interspike intervals were calculated in time interval from 40 to 50 s. For calculation of interspike intervals the threshold potential was set to V_th_ = −40 mV. I_Na_ with slow inactivation.

Figure 6(A–C) shows the bifurcation diagrams representing the dependence of the maximal V_max_ (open circles) and minimal V_min_ (solid circles) values in the transmembrane voltages (panel A), largest/smallest values in [Ca^2+^]_i_ transients (panel B), and the interspike interval (panel C), which is an average delay between any two consecutive APs measured within the time window between 40 and 50 seconds, as the control parameter (steady-state I_stim_) is increased through 1 pA/pF. The voltage diagram in Fig. 6A reveals that for I_stim_ < 0.58 pA/pF, the modeled cell is at a steady-state with relatively small depolarization below −60 mV, where V_min_ = V_max_. At larger stimulus currents (0.58 pA/pF < I_stim_ < 0.79 pA/pF), after the steady state becomes unstable, the cell periodically generates single action potentials (spiking activity). When I_stim_ is increased further, the spike period and the interspike interval decrease, along with the magnitude of the action potentials. The cell transitions from spiking activity to bursting dynamics around I_stim_ = 0.80 pA/pF as indicated by the appearance of additional branches of maximal and minimal amplitudes of the bifurcation diagrams for AP. One can observe that bursting trains near the lower threshold contain large numbers of spikes with varying interspike intervals (Fig. 5D), and that the burst duration increases with decreasing the number of spikes per burst at larger values of I_stim_. The burst duration becomes very long when I_stim_ approaches the threshold value of 0.88 pA/pF (Fig. 6C). Beyond I_stim_ = 0.88 pA/pF, the cell reaches a steady-state with significant depolarization of ~−45 mV. Meanwhile, [Ca^2+^]_i_ transients exhibit behavior other than APs (Fig. 6B). Though the threshold for the onset of periodic oscillations in both AP and [Ca^2+^]_i_ is the same, I_stim_ = 0.58 pA/pF, the noticeable transition from the spiking activity to bursting dynamics of [Ca^2+^]_i_ occurs earlier, at lower I_stim_ = 0.65 pA/pF, then that for the APs. Further increasing I_stim_ to 0.69 pA/pF results in that [Ca^2+^]_i_ reaches a steady state level; the characteristic time evolutions of [Ca^2+^]_i_ are depicted in Fig. 5, panels G–L.

**Figure 7:**
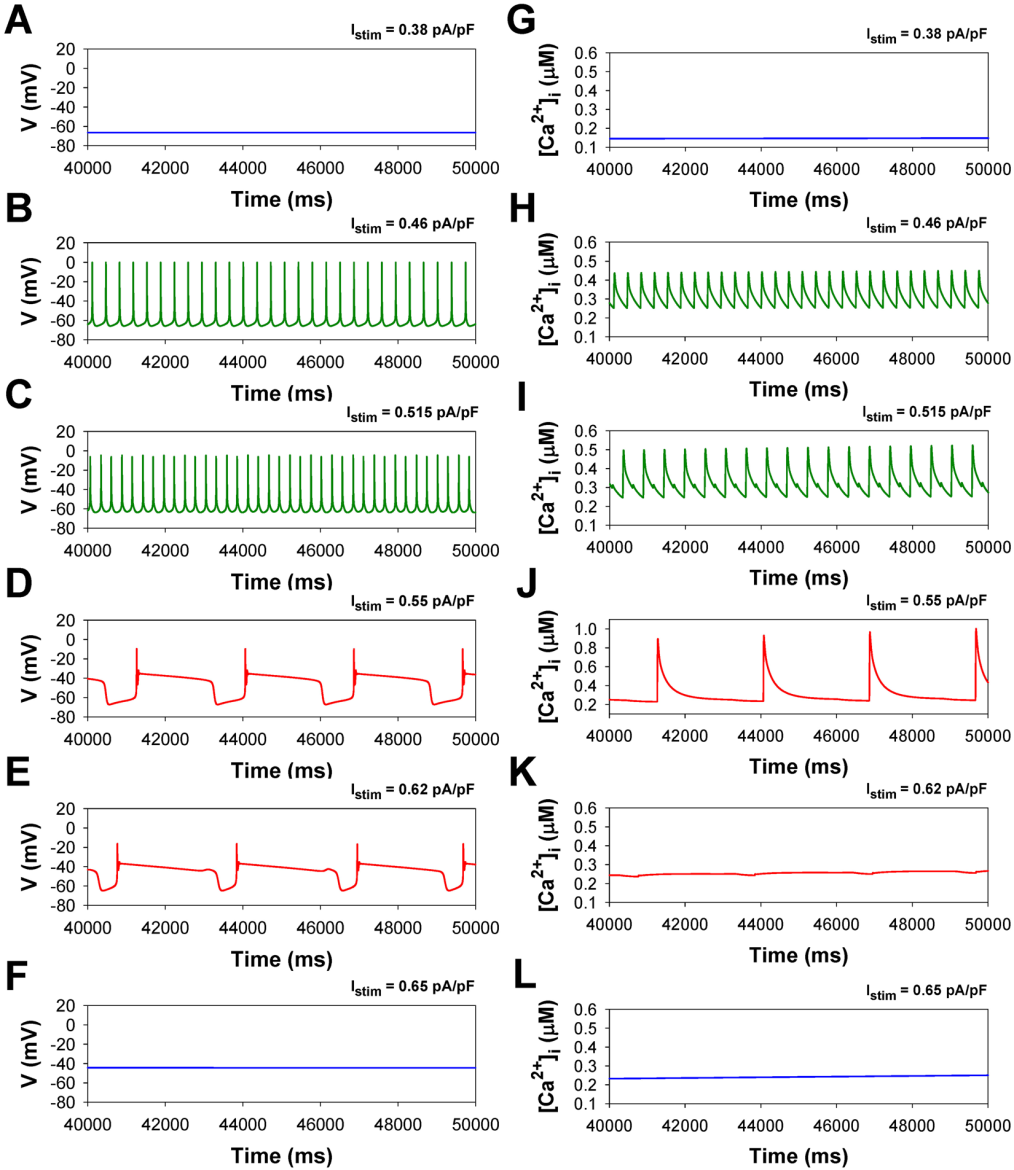
Simulated time series of AP (Panels A–F) and [Ca^2+^]_i_ transients (Panels G–L) in characteristic points of bifurcation diagrams for TNF-α overexpressing mouse ventricular myocytes in Fig. 6 during injection of continuous current: I_stim_ = 0.38 pA/pF (Panels A,G); I_stim_ = 0.46 pA/pF (Panels B,H); I_stim_ = 0.515 pA/pF (Panels C,I); I_stim_ = 0.55 pA/pF (Panels D, J); I_stim_ = 0.62 pA/pF (Panels E,K); I_stim_ = 0.65 pA/pF (Panels F,L). I_Na_ with slow inactivation.

TG mice overexpressing TNF-α demonstrate a larger propensity to instability of APs and [Ca^2+^]_i_ transients in response to continuous stimulation in comparison with WT mice. Figure 6(D–F) represents the bifurcation diagrams for TG mouse cells. Several characteristic time series of the APs and [Ca^2+^]_i_ transients are shown in Fig. 7. For TG mice, the corresponding AP thresholds of I_stim_ for the emergence of spiking activity, transitions from spiking to bursting, and to steady state depolarization are 0.42 pA/pF, 0.53 pA/pF, and 0.64 pA/pF, respectively, which are much lower than those for WT mice. This suggests a larger susceptibility of TG cells to the generation of the action potentials and [Ca^2+^]_i_ transients with the employed stimulation protocol. The analysis of interspike intervals for TG mice also reveals the change in the shape of the bursts compared to ones observed in the wild type cells: unlike WT mice, TG mice cells demonstrate rapidly declining bursts with prolonged depolarization plateaus (Fig. 7D and E). The bifurcation diagram for [Ca^2+^]_i_ transients in TG mice is also different from that in WT mice and demonstrates a different sequence of transitions that occur as I_stim_ is increased (Figs 6E and 7): a stable steady-state, spiking, period-doubling of spikes, high-amplitude periodic [Ca^2+^]_i_ transients, and eventually, a steady-state with a higher [Ca^2+^]_i_ concentration.

**Figure 8:**
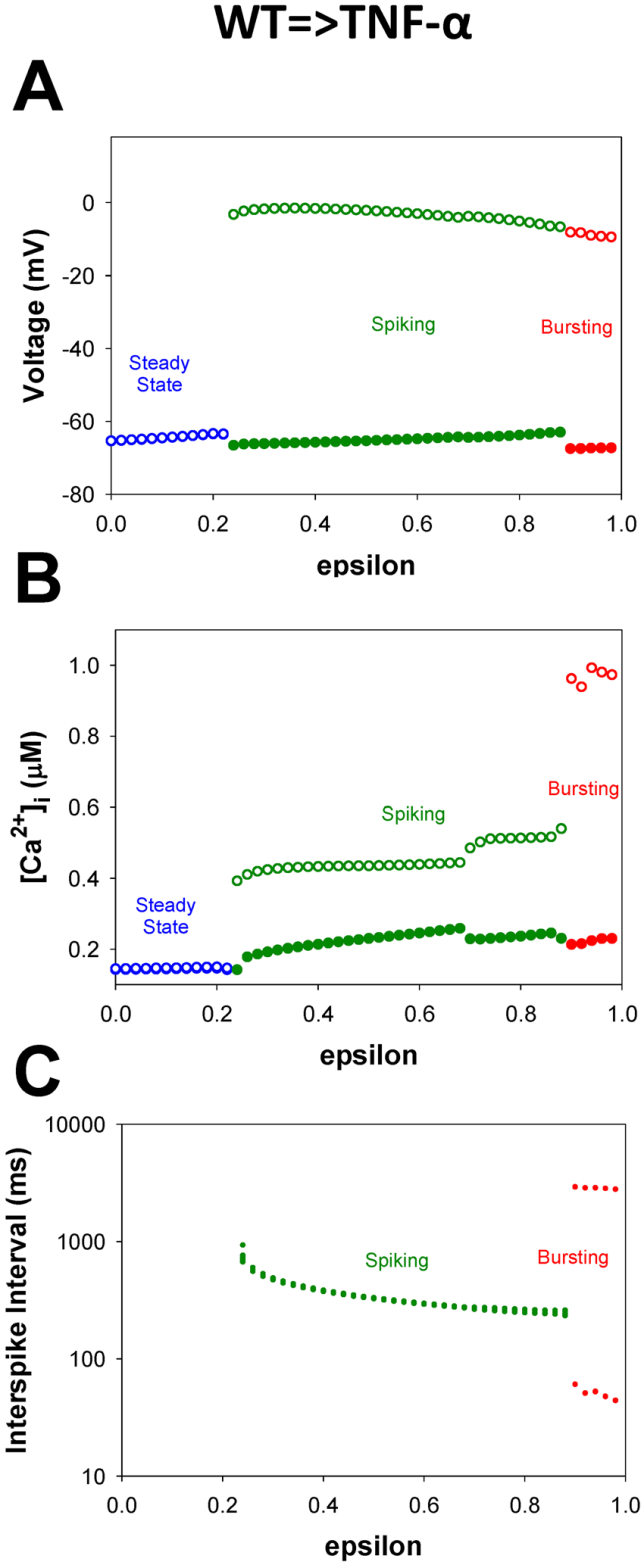
Simulated dependences of AP amplitudes (Panel A), [Ca^2+^]_i_ transients (Panel B), and interspike intervals (Panel C) for mouse ventricular myocytes on the parameter ε during transition from WT to TNF-α overexpressing mouse ventricular myocyte. Steady-state injected current I_stim_ = 0.55 pA/pF. Bifurcation diagrams demonstrate steady-state, periodic activity, and bursting activity both for AP and [Ca^2+^]_i_ transients. AP amplitude, [Ca^2+^]_i_ transients, and interspike intervals were calculated in time interval from 40 to 50 s. For calculation of interspike intervals the threshold potential was set to V_th_ = −40 mV. I_Na_ with slow inactivation.

**Figure 9:**
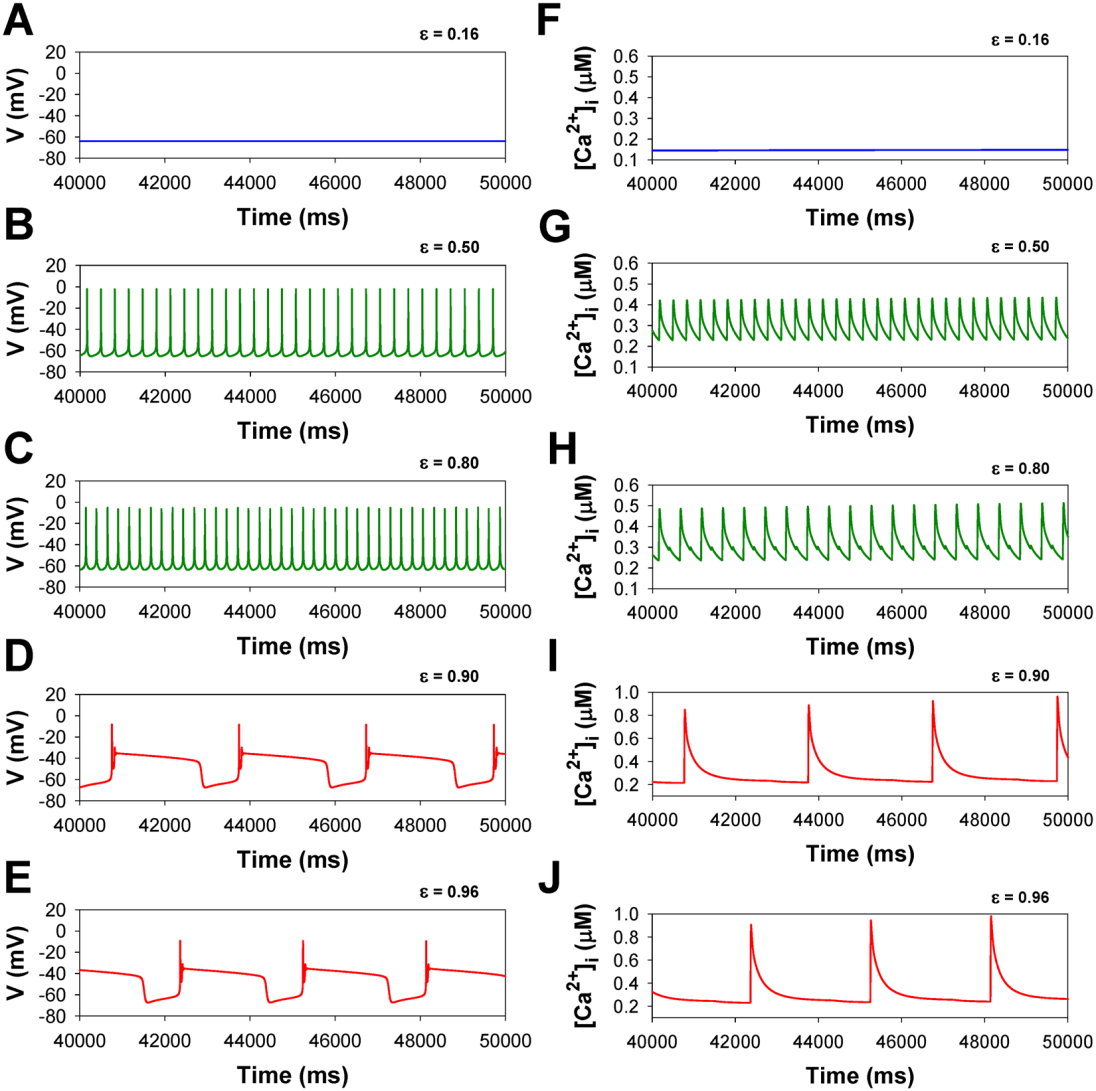
Simulated time series of AP (Panels A–E) and [Ca^2+^]_i_ transients (Panels F–J) in characteristic points of bifurcation diagrams for mouse ventricular myocytes in Fig. 8 at different values of ε during transition from WT to TNF-α overexpressing mouse ventricular myocyte: ε = 0.16 (Panels A,F); ε = 0.50 (Panels B,G); ε = 0.80 (Panels C,H); ε = 0.90 (Panels D,I); ε = 0.96 (Panels E,J);. I_Na_ with slow inactivation.

The behavior of the action potentials, [Ca^2+^]_i_ transients, and interspike intervals for both WT and TG mice as a function of the steady state stimulus current elicited interesting, unexpected dynamics with underlying bifurcations rather than properties evoked by traditional pulse protocol. Nevertheless, this information is still inconclusive to elucidate the key mechanisms differentiating dynamics of the cells at different developmental stages from WT cells to the cells with heart failure. Therefore, we performed bifurcation analysis to infer transition mechanisms from WT to TG cell dynamics by changing all seven principal parameters of the model. As the first approximation, we consider linear changes of all seven parameters from their average values in WT to TG mice. For this purpose, a new consolidated bifurcation parameter ε is introduced so that ε = 0 corresponds to WT cells and ε = 1 corresponds to TG cells. The ε-based bifurcation diagrams for the action potentials, [Ca^2+^]_i_ transients, and interspike intervals are presented in Fig. 8. In these simulations, the value of injected current I_stim_ is set to 0.55 pA/pF. The time series of the APs and [Ca^2+^]_i_ transients at the characteristic points in the bifurcation diagrams are shown in Fig. 9. Comparisons of the ε-based bifurcation diagrams for the AP and [Ca^2+^]_i_ transients (Fig. 8) with those using I_stim_ аs the bifurcation parameter (Fig. 6) infer a noticeable difference, namely: both AP and [Ca^2+^]_i_ transients bifurcate with dynamics changes at the same values of ε, unlike the previous case where the threshold values of I_stim_ for AP and [Ca^2+^]_i_ transients are different.

When ε = 0 (WT mice), both the membrane potential and intracellular [Ca^2+^]_i_ stay at steady states. As ε is increased to 0.24, the steady state loses stability and the AP and [Ca^2+^]_i_ transients exhibit periodic tonic spiking (Fig. 9B). A further increase in ε to 0.70 results in period-doubling bifurcation simultaneously for the AP and [Ca^2+^]_i_ transients. Period-doubling bifurcations, or alternans, which are predicted by this mathematical model, are a typical stage for cardiac cells during development of the heart failure. Alternans are considered as a precursor of more severe rhythm disturbances^12, 13^. Such larger disturbances are also predicted by our simulations, at closer values of ε to the disease state at ε = 0.90, when bursting AP with prolonged plateau emerges and persists through ε = 1.0 corresponding to heart failure. The changes are accompanied by large-amplitude [Ca^2+^]_i_ transients during bursting activity.

To reveal the mechanism of the larger [Ca^2+^]_i_ transients in TG cells as compared to WT cells, we investigated the behavior of the proteins involved in Ca^2+^ dynamics. Our simulations show that the maximum sarcoplasmic reticulum Ca^2+^ concentration in WT mice is larger than in TG mice (1900 µM vs 1600 µM, respectively). However, the opening probability and open time duration of the ryanodine receptors is larger in transgenic mice as compared to wild type mice during bursts. This results in the larger Ca^2+^ release flux in TG cells during Ca^2+^ entry through the L-type Ca^2+^ channels when transmembrane potential exceeds the activation threshold of the L-type Ca^2+^ current (Figs 5C and 7D). Therefore, our mathematical model of transgenic mouse cell demonstrates larger magnitudes of [Ca^2+^]_i_ transients (Figs 5I and 7J). Note that our simulation results are supported by the experimental data by Marx *et al*.^14^, where significantly larger opening probabilities of ryanodine receptors are observed in the failing cells as compared to wild type cells.

As there is a deficit on experimental data on the exact time behavior of changes in seven parameters, which differ in WT from TG mice, in addition to linear dependences, we also explore exponential (faster) dependency for each of the seven bifurcation parameters on ε, while six others are changed linearly (see insert in Supplementary Fig. 2A). The corresponding bifurcation diagrams for the action potentials, [Ca^2+^]_i_ transients, and interspike intervals are presented in Supplementary Figs 1–3, respectively. Remarkably, all bifurcation diagrams indicatively confirm the occurrence of the same qualitative transition stages: from periodic spiking activity to the onset of alternans, and further to bursting dynamics that are observed in experimental studies of progressions to the heart failure.

Our simulations provided necessary data indicating a sensitivity of the disease progression with respect to particular parameters. The analysis of the different types of cellular activity (steady state, spiking, and bursting) and their bifurcations elucidates the functional mechanisms and key contributors causing the transitions. The data presented in Supplementary Figs 1–3 are indicative of the corresponding threshold values of the ε-parameter at which the bifurcation transitions between periodic spiking, period-doubling and bursting occur in both scenarios: with some parameters changing exponentially (faster than others), and ones changing linearly. It infers that the parameters characterizing I_K1_, the conductance G_K1_ and the power index of I_K1_, are the most pro-arrhythmic ones compared to others, because of their exponential exacerbation towards the values observed at heart failure that results in much lower threshold values for ε (0.20 for the conductance and 0.18 for the power index of I_K1_; compared to ε = 0.7 for the linear case) for the emergence of alternans. Our simulations suggest that the changes in I_K1_ are the most pro-arrhythmic factors in the development of the heart failure. Another important candidate is the Ca^2+^ release rate from the sarcoplasmic reticulum, v_3_, the exponential change of which results in the threshold value for ε = 0.62. Both these parameters are implicated as the pivotal ones of the failing hearts which are supported by the known experimental data on the effects of I_K1_ and the SR leak^1^.

**Figure 10:**
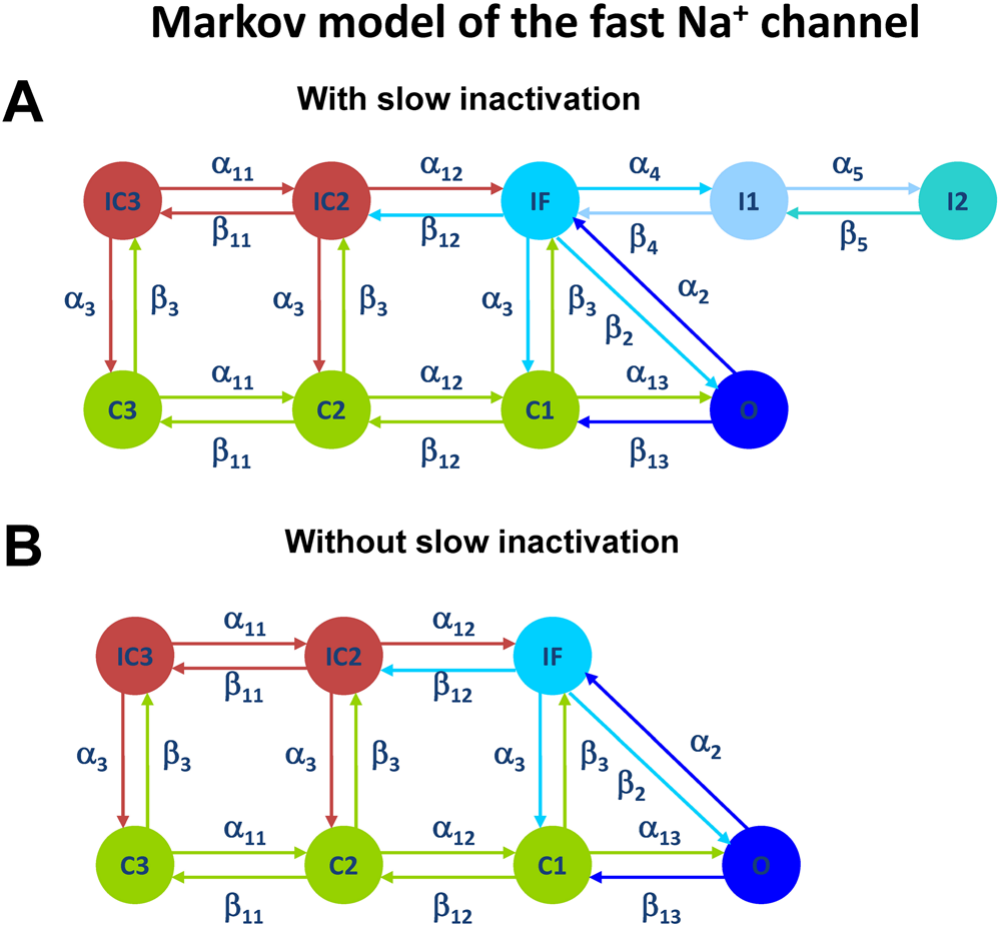
Markov models of the fast Na^+^ current I_Na_ with (Panel A) and without (Panel B) slow component of inactivation. Markov models contain three closed states (C1–C3), one open state (O), two closed-inactivated states (IC2 and IC3), one fast inactivated state (IF), and two slow inactivated states (I1 and I2).

**Figure 11:**
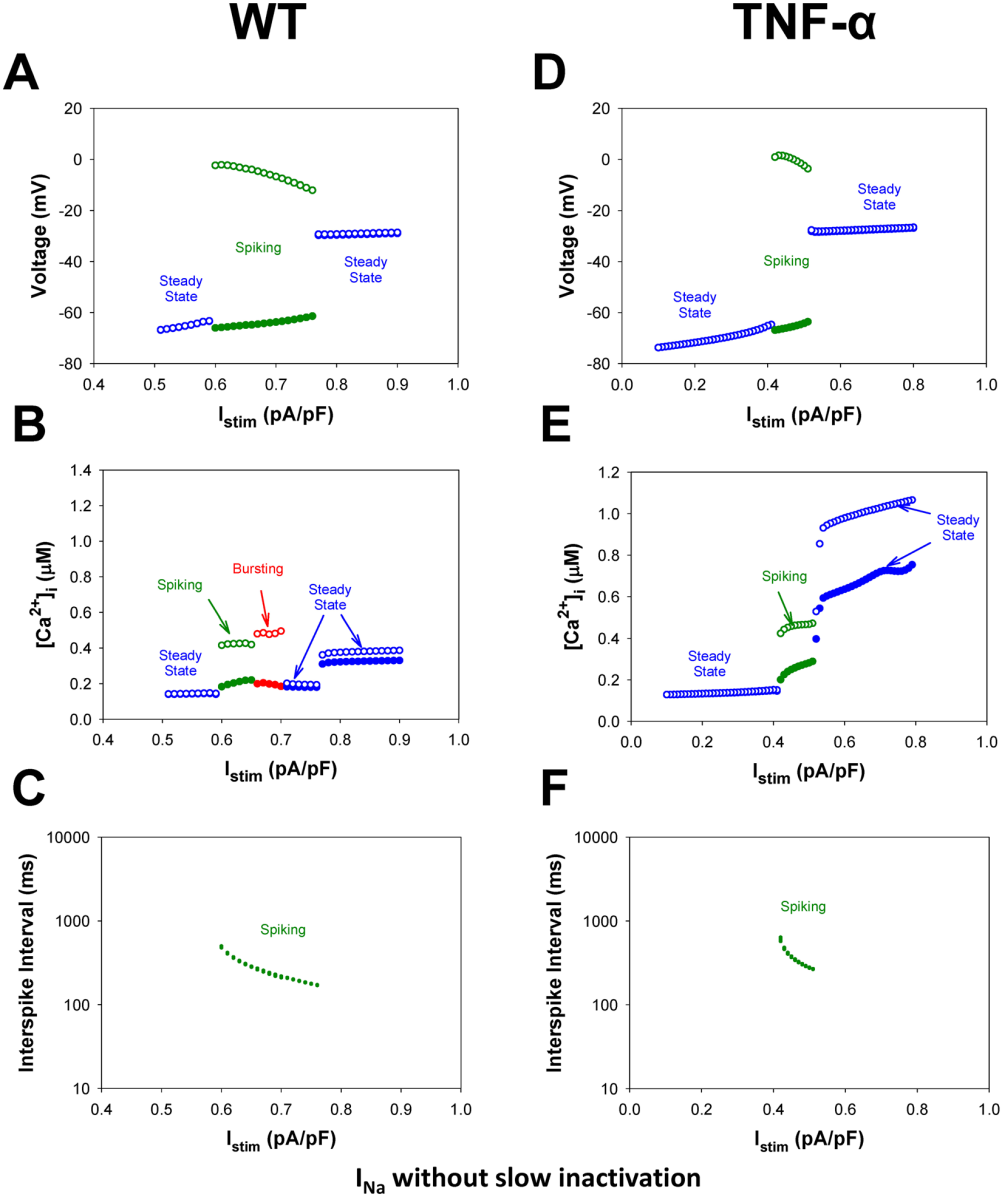
Simulated dependences of AP amplitudes (Panels A,D), [Ca^2+^]_i_ transients (Panels B,E), and interspike intervals (Panels C,F) for the ventricular myocytes from WT (Panels A–C) and TG (Panels D–F) mice on steady-state injected current I_stim_. Bursting activity of AP is absent (Panels A,D). AP amplitude, [Ca^2+^]_i_ transients, and interspike intervals were calculated in time interval from 40 to 50 s. For calculation of interspike intervals the threshold potential was set to V_th_ = −40 mV. I_Na_ without slow inactivation.

**Figure 12:**
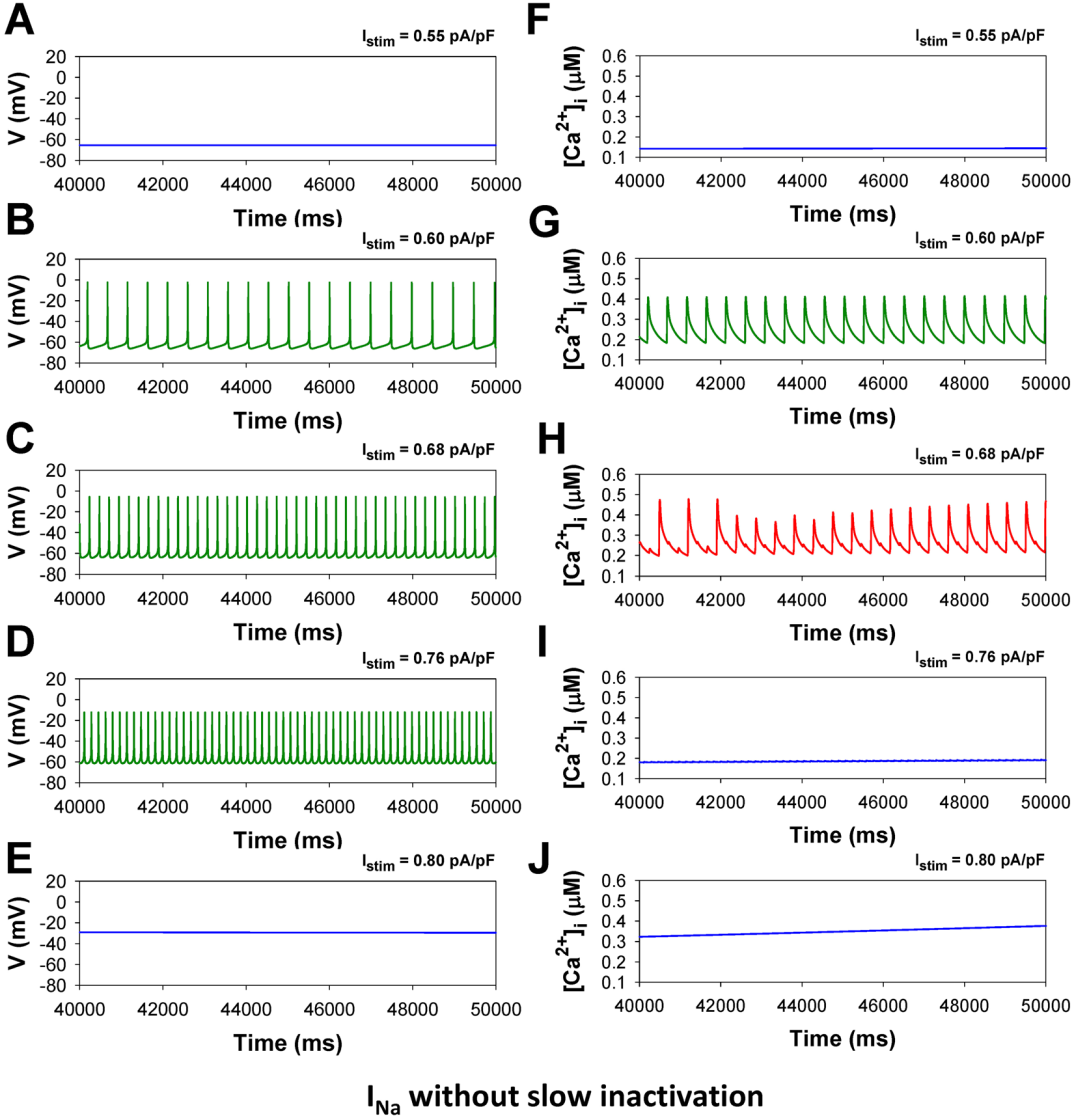
Simulated time series of AP (Panels A–E) and [Ca^2+^]_i_ transients (Panels F–J) in characteristic points of bifurcation diagrams for WT mouse ventricular myocytes in Fig. 11 during injection of continuous current: I_stim_ = 0.55 pA/pF (Panels A,F); I_stim_ = 0.60 pA/pF (Panels B,G); I_stim_ = 0.68 pA/pF (Panels C,H); I_stim_ = 0.76 pA/pF (Panels D,I); I_stim_ = 0.80 pA/pF (Panels E,J). I_Na_ without slow inactivation.

In addition, we reveal the major contributor to the generation of bursting activity in the mouse ventricular myocytes. While generic cellular mechanisms giving rise to periodic activity beginning with Andronov-Hopf bifurcations followed by period-adding and doubling cascades are well-interpreted and investigated, transitions leading to the onset of bursting activity in cardiac cells through global bifurcations are less known^15–19^. Our simulation analysis substantiates the role of the slow inactivation mechanism of the fast Na^+^ current in the buildup of bursting activity in the mouse ventricular myocyte. The analysis of the maps of the fast variable (transmembrane voltage V) versus other variables revealed that the only variable that changes slowly during bursts is the state occupancy of the slow inactivation of I_Na_, I1 (inactivated state I2 is not significantly involved in the dynamics of I_Na_). Therefore, we removed the slow inactivation of I_Na_ from the Markov model and investigated the resulting model behavior (Markov models for the fast I_Na_ with and without slow components of inactivation are presented in Fig. 10). Figure 11 presents the bifurcation diagrams for the action potential, [Ca^2+^]_i_ transients, and interspike intervals for the fast Na^+^ current without slow inactivation states in the corresponding Markov model plotted against the steady state stimulus I_stim_ for WT (Fig. 11A–C) and TG mice (Fig. 11D–F). The AP bifurcation diagrams in Fig. 11A and D clearly show two transitions on the route: from the steady state to the spiking activity, and further to another steady state with significant depolarization. However, TG mice demonstrate smaller I_stim_ for the onset of spiking activity and shorter interval for I_stim_ when the spiking occurs. In addition, Fig. 11C and F demonstrates nearly an order of magnitude decrease of the interspike intervals during the spiking window in the diagram for both WT and TG mice. Meanwhile, the bifurcation diagram for [Ca^2+^]_i_ transients in WT mice (Fig. 11B) shows that bursting activity emerges and develops similarly to that in cells with intact slow inactivation of I_Na_ (compare to Fig. 6B). Figure 12 shows time series for several characteristic points in the bifurcation diagrams in Fig. 11 for WT mice (similar behavior is observed in TG cells, but with slightly different characteristics, and bursting [Ca^2+^]_i_ was not observed).

**Figure 13:**
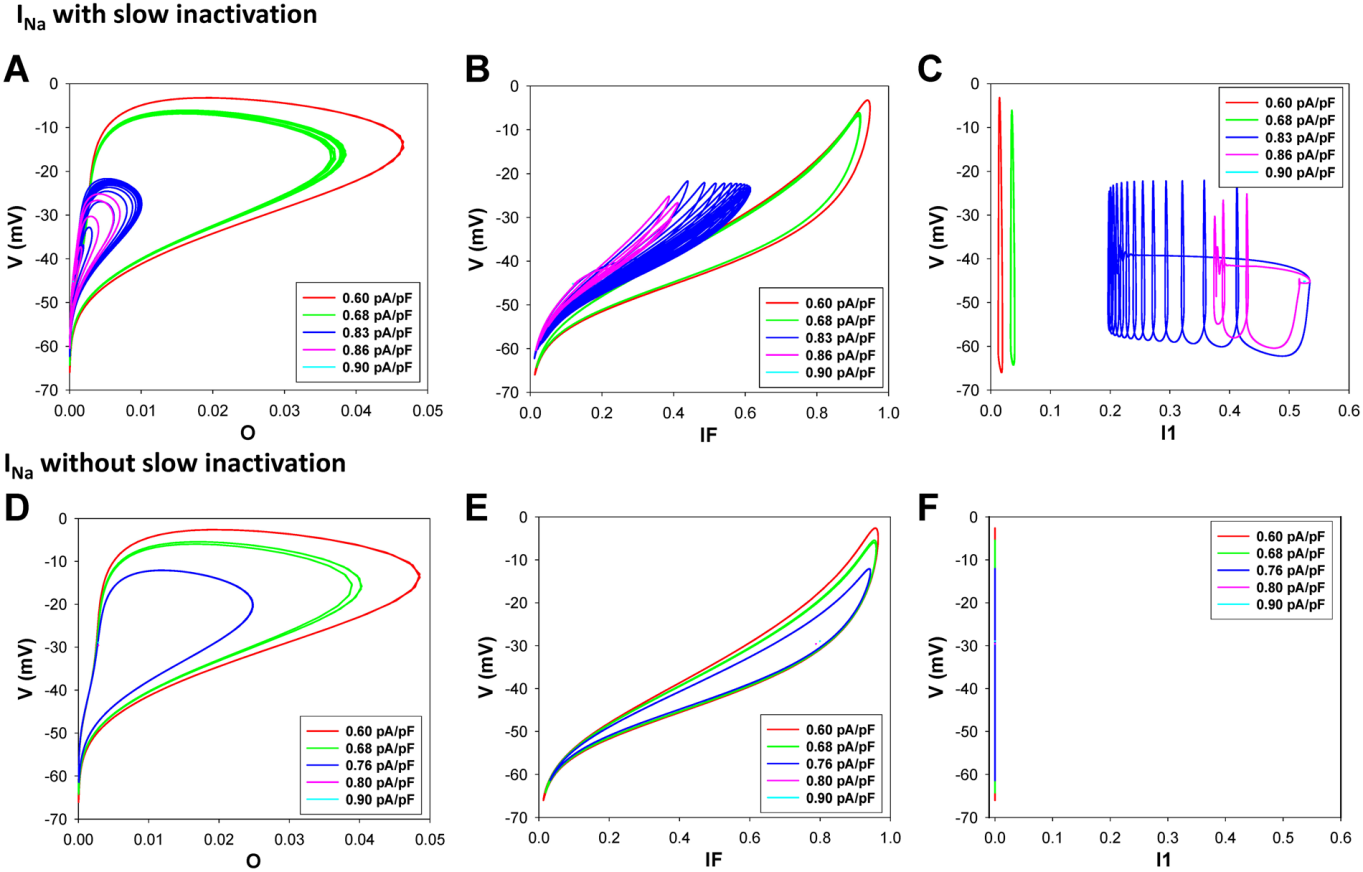
Simulated maps of transmembrane voltage V versus probabilities of the fast Na^+^ channels to be in open (O; Panels A,D), fast inactivated (IF; Panels B,E), and slow inactivated (I1; Panels C,F) states for different values of injected steady-state current I_stim_ in WT cells. I_Na_ with (Panels A–C) and without (Panels D–F) slow inactivation.

Figure 13 depicts several phase portraits mapping the transmembrane voltage V dynamics against dynamics of the occupancies of the open state O, fast-inactivated state IF, and of the major slow-inactivated state I1, with and without slow inactivation of I_Na_ for the several continuous I_stim_ values corresponding to robust periodic activity, alternans, bursting and steady states for WT mice. It becomes clear from the phase portraits that bursting activity is due to slow inactivation of the sodium current I_Na_ in mouse ventricular cells (Fig. 13C). This is not the case for I_Na_ without slow inactivation where dynamics morph first from periodic activity towards alternans through period doubling, and after to steady states only (occupancies for the I1-state vanish, Fig. 13F).

## Discussion

This modeling study elucidates key mechanisms of transition in mouse ventricular myocytes from the normal to failing state that is described as a sequence of bifurcations that occur as multiple control parameters change from regular to pathological values. The transition includes several stages such as steady states, periodic spiking activity, alternans with double periods, and bursting activity. The transition between the stable steady state and tonic spiking occurs through a supercritical Andronov-Hopf bifurcation. While we simulated transitions under the condition of injection of steady current, they demonstrate activities, which are quite similar to those reported in experimental studies of heart failure in a diverse set of species^12, 13^. We found that that the threshold stimulus-current triggering bursting dynamics in [Ca^2+^]_i_ transients is lower than that for the action potentials. Wild type cells and cells from mice overexpressing TNF-α demonstrate different patterns of bursting behavior. It is shown that the slow inactivation of the fast Na^+^ current is the major cause of the bursting activity.

Despite several, biologically plausible, mathematical models which have been recently developed for WT ventricular myocytes and failing cardiomyocytes of different species^7, 8^, there is still a dire need for a comprehensive mathematical model that can describe and predict characteristic transition behaviors of the action potential and [Ca^2+^]_i_ transients during the developmental stages towards heart failure. This paper proposes a new approach to studying such transitions to the diseased state on bifurcation routes/pathways in a multi-dimensional space of the control parameters of the mathematical model. The proposed approach describes the key transition stages (alternans, bursting, and high-amplitude [Ca^2+^]_i_ transients) towards heart failure^20^. The results of simulations, elucidating different progressions of the bifurcation parameters from the healthy to the failing values (observed at heart failure), *de-facto* validate the robustness of the model approach proposed for the prediction of multiple transition stages towards the diseased state. In future studies, the modeling method can also be supplemented with experimentally identified bifurcation values that would allow us to reveal and predict the characteristic dynamic features of the cells during the development towards heart failure, whether these be regular alternans, transitioning to complex activity such as regular or irregular bursting. In particular, other experimental mouse models of heart failure (such as myocardial infarction or transverse aortic constriction) can be simulated using the proposed method of multiparametric bifurcations. In those cases, we need to define the set of parameters that change from WT mouse model to the mouse model with heart failure and investigate their effects on the behavior of the action potential and [Ca^2+^]_i_ transients.

Note that the stimulation of cardiac cells with a constant injected current (or prolonged injected current pulses) was recently employed in transgenic mice expressing channelrhodopsin-2 (ChR2)^11^. Irregular heart beats and action potential propagation block were observed upon continuous stimulation of ChR2, resulting in the inward continuous current. Our novel stimulation protocol can provide some insights into the mechanisms of different optically generated behaviors of the mouse heart.

Our modeling study clearly reveals that the slow inactivation of the fast Na^+^ current underlies the onset of composite bursting dynamics in the mouse ventricular myocyte. Our simulations have displayed that the elimination of the slow component of inactivation of the fast Na^+^ current terminates bursting activity in ventricular cells. While intact slow inactivation of I_Na_ promotes bursting activity, it can also provide significantly lower cellular depolarization at relatively large I_stim_, compared to the values observed in the cells without slow inactivation. This conclusion can be verified by further experiments. The fast Na^+^ current is responsible for the upstroke of the cardiac action potential in multiple species. Dysfunctions of these channels can lead to lethal arrhythmias and heart failures. Experimental data discloses an involvement of dysfunctional slow inactivation of I_Na_ into the development of pro-arrhythmic activity and heart failure^21–23^. Both enhanced and reduced slow inactivation of I_Na_ resulted in pro-arrhythmic phenotype^21, 23^. Our mathematical modeling also supports this conclusion: elimination of slow inactivation of I_Na_ results in larger cellular depolarization, whereas intact slow inactivation of I_Na_ promotes bursting activity in cardiac cells.

While the cellular origin of pro-arrhythmic behavior is well documented^24^, the intercellular coupling also plays an important role in arrhythmia development. In particular, the increased propensity to arrhythmias is observed in transgenic mice overexpressing TNF-α^4^. We used a mathematical model of mouse ventricular myocytes from wild type and transgenic mice overexpressing TNF-α to demonstrate that the slowing conductance of the action potential propagation in transgenic mice leads to widening the vulnerable window corresponding to sustained unidirectional action potential propagation^7^.

Despite that our comprehensive mathematical models describe experimentally observed transient behaviors from the normal to the failing mouse hearts, the approach has several limitations. First, we employed currently available comprehensive mathematical models for the wild type and transgenic mouse ventricular cells overexpressing TNF-α. We did not investigate other mouse models of heart failure, such as myocardial infarction or transverse aortic constriction, for which comprehensive mathematical models are not available. Second, while our mathematical models demonstrate [Ca^2+^]_i_ alternans in multicellular tissue^7^ and upon stimulation with continuous current (Figs 7,9 and 13), we were unable to reproduce [Ca^2+^]_i_ alternans observed recently by Hammer *et al*.^25^ upon pulsed stimulation in isolated mouse ventricular myocytes. Third, we did not implement a description of the late Na^+^ current, I_NaL_^26^, the inactivation of which is very slow, ~0.6 s at 37 °C. Nevertheless, its influence is partially accounted for by the background Na^+^ current in the presented model. Fourth, we obtained our results using a mathematical model of mouse ventricular myocytes. The results can be different for larger mammals, which have a different action potential shape and underlying ionic currents.

Thus, in this paper, we proposed a novel method of stimulation of the ventricular myocyte by application of a constant current. We showed that this stimulation method leads to a wider range of different cell activities (steady-state, periodic activity, bursting activity, increased cellular depolarization), as well as demonstrated that the cellular activity can be represented as a sequence of bifurcations from wild type cell to the failing cell. Finally, the slow component of inactivation of the fast sodium current was revealed to be the pivotal factor causing the onset of bursting activity in mouse ventricular myocytes.

## Electronic supplementary material

Supplementary Material
