# Supplementary material for: Bursting dynamics in the normal and failing hearts
Source: Sci Rep. 2017 Jul 19;7:5927. doi: 10.1038/s41598-017-05198-z (PMC5517618; doi:10.1038/s41598-017-05198-z)
Supplement: Supplementary file 1 — Supplementary Material [file 41598_2017_5198_MOESM1_ESM.pdf]

Bursting dynamics in the normal and failing hearts

[1]Vladimir E. Bondarenko and [1,2]Andrey L. Shilnikov

[1]Department of Mathematics and Statistics and Neuroscience Institute,
Georgia State University, 30 Pryor Street, Atlanta, GA 30303;
[2]Institute for Information Technologies, Mathematics and Mechanics, Nizhni Novgorod State University, Gagarin Av. 23, 606950 Nizhni Novgorod, Russia

Supplementary Material

Address for Correspondence:

Dr. Vladimir E. Bondarenko
Department of Mathematics and Statistics
Georgia State University
30 Pryor Street, 732 COE
Atlanta, GA 30303-3083

Telephone: 404-413-6440
FAX: 404-413-6403
Email: vbondarenko@gsu.edu

Supplementary Figure Legends

Supplementary Figure 1: Simulated dependences of AP amplitudes for mouse ventricular myocytes on the parameter ε during transition from WT to TNF-α overexpressing mouse ventricular myocyte. Steady-state injected current I_{stim} = 0.55 pA/pF. Data in **Panel A** is shown for the linear change of 7 major parameters. Data in other **Panels** is shown for linear change of 6 major parameters and exponential change of the 7th parameter (SR Ca^{2+}-ATPase maximum pump rate v_3, **Panel B**; scaling factor for Na^+/Ca^{2+} exchanger k_{NaCa}, **Panel C**; maximum transient outward K^+ current conductance $G_{Kto,f}$, **Panel D**; maximum ultrarapidly delayed-rectifier K^+ current conductance G_{Kur}, **Panel E**; maximum time-independent K^+ current conductance, **Panel F**; power index of the time-independent K^+ current, **Panel G**; linear component of the time-independent K^+ current, **Panel H**). AP amplitude, $[Ca^{2+}]_i$ transients, and interspike intervals were calculated in time interval from 40 to 50 s. For calculation of interspike intervals the threshold potential was set to V_{th} = –40 mV. I_{Na} with slow inactivation.

Supplementary Figure 2: Simulated dependences of $[Ca^{2+}]_i$ transients for mouse ventricular myocytes on the parameter ε during transition from WT to TNF-α overexpressing mouse ventricular myocyte. Notations are the same as in Supplementary Figure 1. Insert in **Panel A** shows linear (solid line) and exponential (dashed line) changes of bifurcation parameters between their maximum and minimum values.

Supplementary Figure 3: Simulated dependences of interspike intervals for mouse ventricular myocytes on the parameter ε during transition from WT to TNF-α overexpressing mouse ventricular myocyte. Notations are the same as in Supplementary Figure 1.

Supplementary Figure 1

WT=>TNF-α

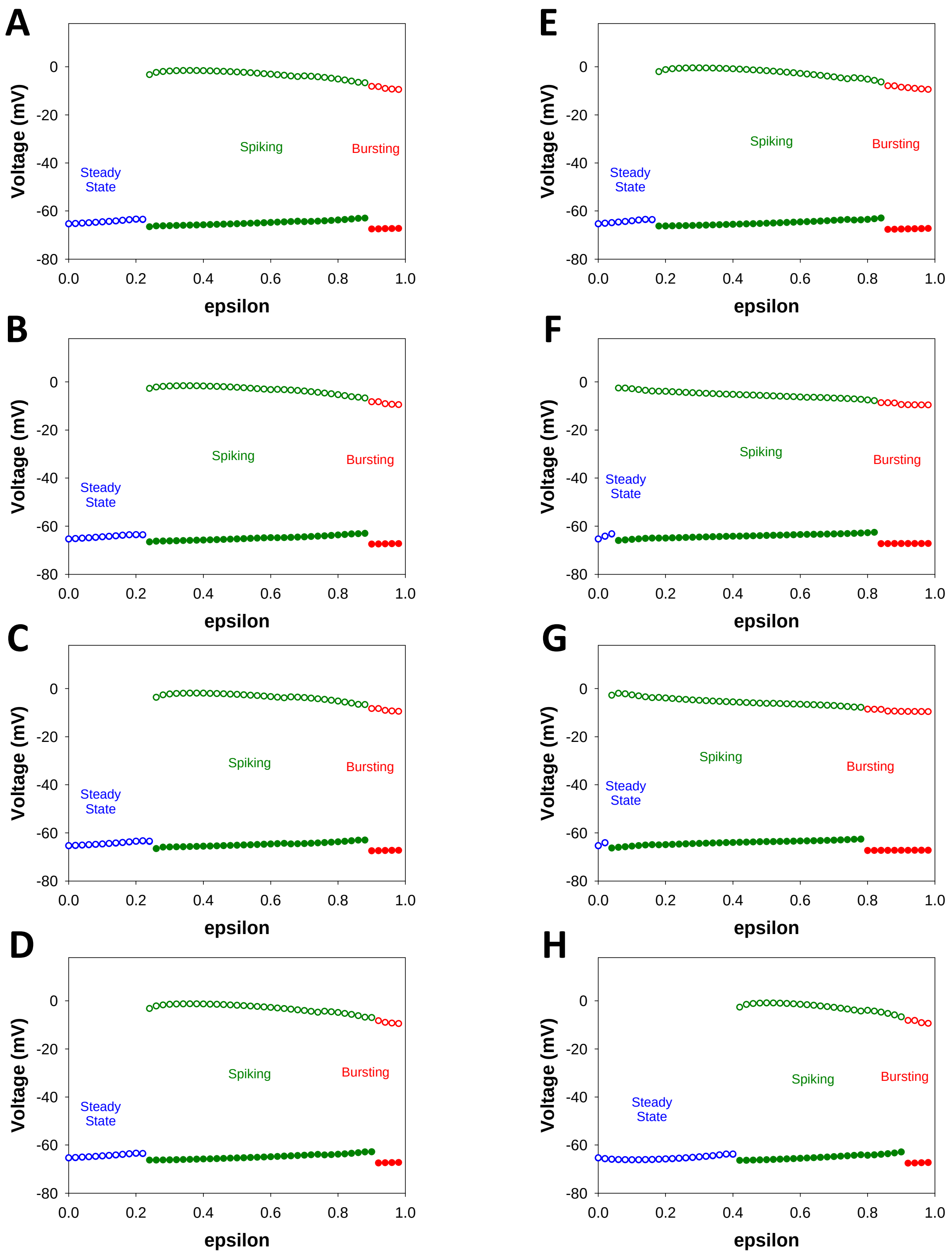

Supplementary Figure 2

WT=>TNF-α

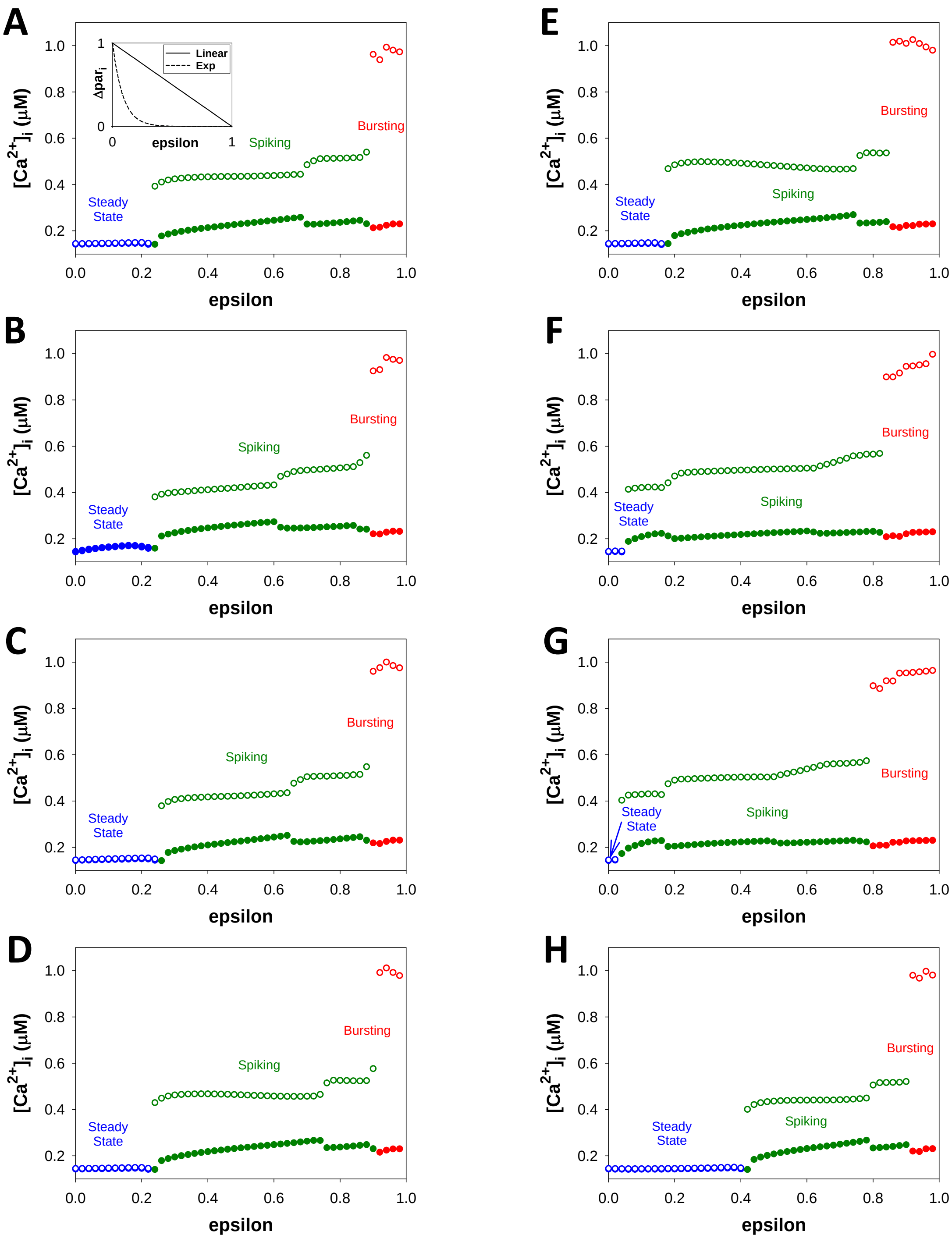

Supplementary Figure 3

WT=>TNF-α

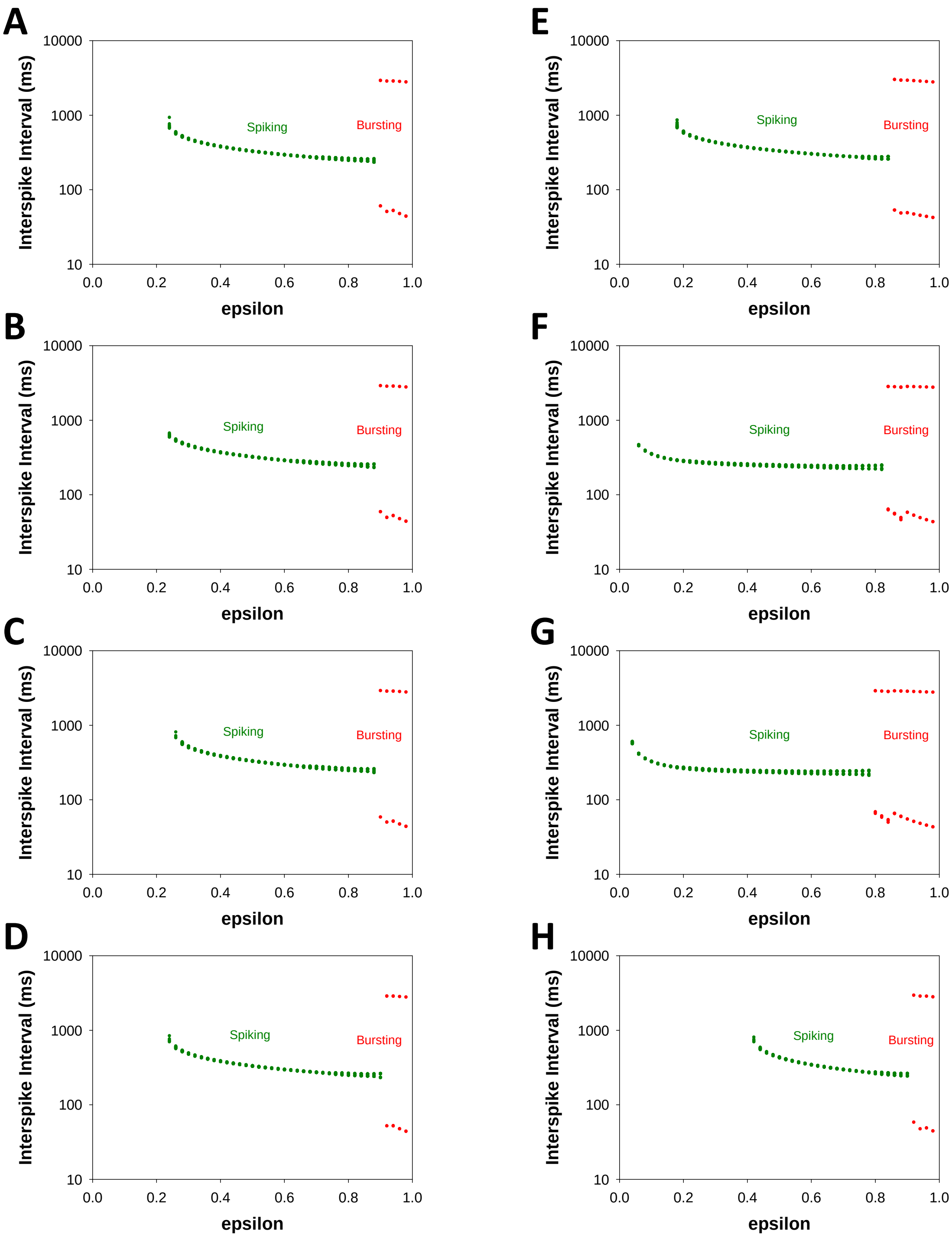

Supplementary Table 1. Differences between WT and TG mouse ventricular myocytes

Model Parameter	WT	TG
Maximum transient outward K^+ current conductance	$G_{Kto,f} = 0.3846$ mS/µF	$G_{Kto,f} = 0.2004$ mS/µF
Maximum ultrarapidly delayed-rectifier K^+ current conductance	$G_{Kur} = 0.3424$ mS/µF	$G_{Kur} = 0.2362$ mS/µF
Scaling factor for Na^+/Ca^{2+} exchanger	$k_{NaCa} = 234.24$ pA/pF	$k_{NaCa} = 351.36$ pA/pF
Time-independent K^+ current	$I_{K1} = 0.3397\left(\frac{[K^+]_o}{[K^+]_o + 210.0}\right)\left[\frac{V - E_K}{1 + e^{0.0448(V - E_K)}}\right] + 0.0193V$	$I_{K1} = 0.3224\left(\frac{[K^+]_o}{[K^+]_o + 210.0}\right)\left[\frac{V - E_K}{1 + e^{0.0474(V - E_K)}}\right] + 0.0184V$
SR Ca^{2+}-ATPase maximum pump rate	$v_3 = 0.315$ µM/ms	$v_3 = 0.1575$ µM/ms
